# Age-related processing delay reveals cause of apparent sensory excitability following auditory stimulation

**DOI:** 10.1038/s41598-017-10696-1

**Published:** 2017-08-31

**Authors:** Márta Volosin, Zsófia Anna Gaál, János Horváth

**Affiliations:** 1 Institute of Cognitive Neuroscience and Psychology, Research Centre for Natural Sciences, Hungarian Academy of Sciences, Budapest, H-1117 Magyar Tudósok körútja 2., Hungary; 2 Eötvös Loránd University, Faculty of Education and Psychology, Budapest, H-1075 Kazinczy utca 23–27., Hungary; 3 University of Leipzig, Institute of Psychology, Cognitive and Biological Psychology, Leipzig, D-04109 Neumarkt 9–19, Germany

## Abstract

When background auditory events lead to enhanced auditory event-related potentials (ERPs) for closely following sounds, this is generally interpreted as a transient increase in the responsiveness of the auditory system. We measured ERPs elicited by irrelevant probes (gaps in a continuous tone) at several time-points following rare auditory events (pitch glides) in younger and older adults, who watched movies during stimulation. Fitting previous results, in younger adults, gaps elicited increasing N1 auditory ERPs with decreasing glide-gap separation. N1 increase was paralleled by an ERP decrease in the P2 interval. In older adults, only a glide-gap separation dependent P2 decrease, but no N1-effect was observable. This ERP pattern was likely caused by a fronto-central negative waveform, which was delayed in the older adult group, thus overlapping N1 and P2 in the younger, but overlapping only P2 in the older adult group. Because the waveform exhibited a polarity reversal at the mastoids, it was identified as a mismatch negativity (MMN). This interpretation also fits previous studies showing that gap-related MMN is delayed in older adults, reflecting an age-related deterioration of fine temporal auditory resolution. These results provide a plausible alternative explanation for the ERP enhancement for sounds following background auditory events.

## Introduction

When absorbed in a task, task-irrelevant stimuli seem to fade into the background. Moments of such immersion still do not provide a complete isolation from the stimulus environment: sudden changes in the acoustic background still capture our attention, even if they are irrelevant to the ongoing behavior. By “opening up” the sensory system, such involuntary re-allocations of attention (*distraction* ^1^) allow the acquisition of information that may initiate the re-evaluation of behavioral goal priorities, and thus lead to a change or discontinuation of the ongoing behavior. The sound of an approaching vehicle on the street may compel us to look up from a smartphone screen and take evasive action. Finding proper balance between the ability to focus on one’s immediate behavioral goals, and the ability to be distracted by potentially goal-changing sensory information is crucial for successful adaptation. Older adults are often characterized as being less able to inhibit the processing of task-irrelevant information and therefore more susceptible to distraction than younger adults^2–6^. This may be interpreted as a shift in the attention-distraction balance. Distraction, however, is not a unitary phenomenon^7, 8^, and impacted performance attributed to higher distractibility may result from changes in various functions contributing to the attention-distraction balance. For example, a lower sensory threshold^9^ that allows intrusions of stimuli with low potential to be behaviorally relevant (a more “open” sensory state) may impact overall performance because distraction-reorienting cycles occur too often. Decreased performance may, however, also result from increased processing times: in older adults more time may be needed for the completion for an involuntary attention switch, whereas re-orienting may take longer in children^10^. The goal of the present study was to compare the persistence of a more “open” (distracted) sensory state induced by background auditory changes in younger and older adults. The duration of distracted state was measured by probing the capability to process auditory events (as reflected by auditory event-related potentials – ERPs) at several time points after distracter onset.

Rapid changes in auditory stimulation (e.g. sound onsets, pitch changes, or gaps in continuous sounds, referred to as *auditory events* in the following) elicit a sequence of characteristic ERP waveforms, which reflect various stages of auditory information processing^11, 12^. The late part of the auditory ERP, specifically the N1 and P2 waveforms can be utilized to probe the processing capability of the auditory system at a given moment. N1 peaks fronto-centrally at around 100 ms, while P2 exhibits a central peak typically in the 160–200 ms range following the auditory event. Although initially these waveforms were regarded as a unitary phenomenon (the “vertex potential”^13, 14^), later studies demonstrated their independence (for a review, see Crowley and Colrain^15^). Both waveforms are generated (at least in part) in the auditory areas of the temporal cortex^16–18^, and reflect the physical parameters of the stimulation. Whereas there is a consensus on that N1 reflects auditory change detection^14, 19^, the functional role of P2 is poorly understood, with suggestions including stimulus evaluation mechanisms^15^, generators related to conscious perception thresholds^20^ or perceptual learning^21, 22^.

Importantly, N1 (and possibly P2) amplitude also reflects the readiness of the auditory system to process incoming stimulation. It is well-known, for example, that attentional state influences N1 amplitude: N1 is enhanced when it is elicited by events in the focus of attention^11, 23–26^, whereas it is attenuated when elicited by events presented during a period of distraction^27, 28^. In the present study, we exploited this to assess the duration of a distracted state by measuring the amplitudes of N1s elicited by probe-events following distracters at several time points. Although a number of ERPs may overlap P2, several studies found enhanced P2 amplitudes in case when tones were attended actively compared to passive listening conditions^28–30^ (but see Hillyard *et al*.^11^).

The most efficient auditory distracters are rare, unpredictably occurring, or salient events^9, 31–33^. Such sound events typically elicit enhanced N1s, mismatch negativity (MMN), and P3a. Whereas the negativities reflect auditory change detection processes^12, 19^, P3a is generally interpreted as a reflection of attentional orienting towards the stimulus^34^. To investigate the duration of a distracted state, we adapted the passive version of the continuous stimulation paradigm introduced by Horváth and Winkler^28^. In their paradigm, a continuous tone was presented, which alternated between two pitch levels by occasional, randomly timed, quick glissandos (glides). Short silent periods (gaps) were also randomly inserted into the tone. Whereas gaps occurred frequently (on average once every 2.6 s), glides were rare (on average once every 9.75 s). In the *active* version of the paradigm, participants’ task was to respond to gaps by pressing a button. Due to their infrequency and unpredictability, the glides functioned as distracters in these paradigms: Horváth^27^, Volosin, Grimm, and Horváth^35^ found that rare glides elicited a higher N1 than frequent glides (and possibly an MMN), but no P3a. Importantly, gaps following rare glides in 150 ms elicited lower-amplitude N1s in comparison to gaps following glides by 650 ms (see also Schröger^36^), or in comparison to gaps without closely preceding glides^28^. This impacted auditory processing suggests that 150 ms after the distracter onset the task-optimal attention set for gap-detection was not yet reinstated. Although evidence on duration of allocation of attention in auditory modality is scarce, this is in line with studies suggesting that attention switch occurs between the time range of N1 and P3, starting at about 130 ms and lasting until about 300 ms^37, 38^.

When the same stimulation was administered to participants who watched a self-selected movie and ignored the tone (*passive* version), gap-related N1 amplitudes showed the opposite pattern: gaps following glides in 150 ms elicited enhanced N1s in comparison to gaps not closely preceded by other events, that is, 150 ms after a glide, auditory processing was enhanced. Horváth and Winkler^28^ suggested that the enhancement reflected attention capture by the rare glide, which diverted attention from the movie to the auditory stimulation. Whether the enhancement was caused by attentional orienting, or by other mechanisms, is unclear. Similar N1 differences were also reported when identical tones followed each other in short (<400 ms) time compared to those at longer separations^39–44^: tones following in shorter (than 400 ms) intervals elicited higher N1s than those following with larger separations. These results can be interpreted by assuming a short-term facilitatory effect following tone onset^40^, or a more complex interaction of facilitation and inhibition. According to the *latent inhibition* model^41, 43^, tone onsets cause a general facilitation in the auditory cortex, which also spreads to neural structures inhibiting N1-generation. Due to their temporally different unfolding, facilitation dominates till about 400 ms, after which inhibition becomes dominant.

Aging is associated with higher susceptibility to distraction, manifested as a decreased ability to filter sensory input^45, 46^ and to inhibit the processing of task-irrelevant pieces of information^5, 47–49^. Age-related sensory ERP enhancements are also often attributed to decreased inhibition of incoming stimulation (e.g. Chao and Knight^50^) which result for example in enhanced slowing in reaction times to distracters^3, 6^. In the present context, we hypothesized that an increased distractibility, or a decreased ability to inhibit the processing of task-irrelevant, background auditory events would be manifested in a longer-lasting enhanced responsiveness to probe events following a distracter. Accordingly, the enhancement of N1 (and possibly P2) would be observable for longer glide-gap separations in older than in younger adults.

To test this hypothesis, in the present study, glide-gap separation was varied in the continuous stimulation paradigm: rare glides could precede gaps in 150, 250 or 650 ms, but gaps without closely preceding glides (“gap only” events), and glides without closely following gaps (“glide only” events) also occurred. By subtracting the “glide only” ERPs from “glide-and-gap” ERPs, the gap-related ERP could be assessed separately from the preceding glide-related waveform. “Gap only” ERPs served as a baseline for assessing ERP enhancements. In contrast with previous studies which used a procedure relying on the assumption that the range of the between-stimulus jitter was sufficiently large to allow the estimation and subtraction of the ERPs related to the preceding tone^51^, the present paradigm allowed a simple subtraction of ERPs related to the preceding rare glide. In assessing ERP enhancements, one has to take into account that ERPs may be different between groups per se. Indeed, numerous studies show that in older adults, late auditory ERPs elicited by sound onsets tend to be larger^52–54^ than in younger adults, while gap-related ERPs were found to be smaller in older adults^55, 56^. Because of this, ERP enhancements were expressed as amplitude proportions of the respective gap-only ERPs separately in the two age groups.

## Results

### Gap-related ERPs – hypothesis-driven analysis

Following the exclusion of the artifact-contaminated epochs, individual ERPs were averaged separately for the two groups for each gap position. The mean epoch numbers (and their standard deviations) in the younger adult group for the 150 ms and 650 ms gaps were 81 (SD = 3.21 and 3.47, respectively), for the 250 ms gaps 82 (SD = 2.28) and for gap only events 1427 (SD = 46.31). “Glide only” events included 244 (SD = 7.03) epochs on average. In the older adult group the mean epoch numbers for 150 ms, 250 ms and 650 ms gaps were 81 (SD = 4.24; 3.66 and 4.99, respectively). “Gap only” events were averaged from 1408 (SD = 69.5), “glide only” events from 241 (SD = 12.37) epochs.

**Figure 1:**
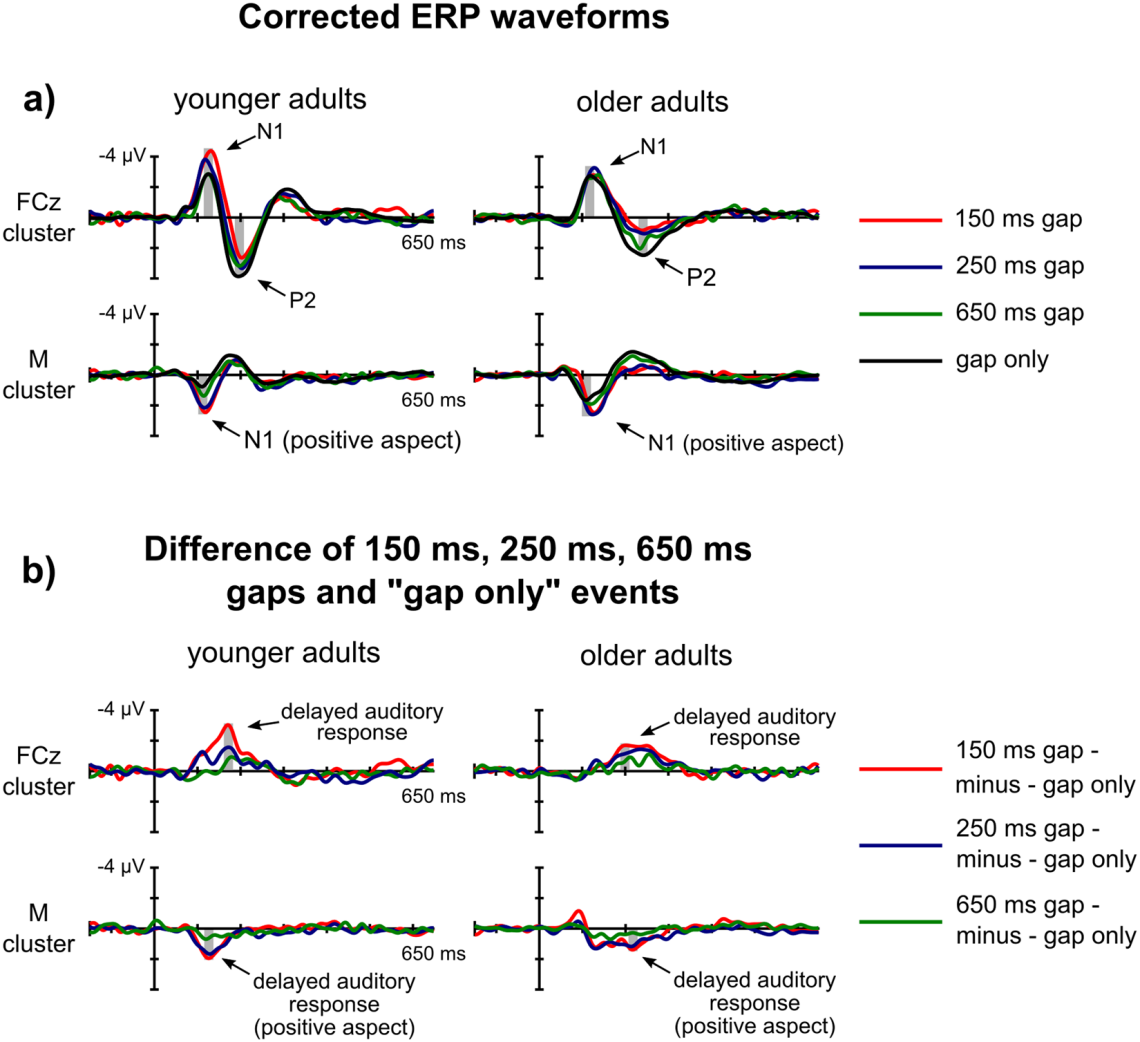
Group-mean gap-related ERPs. Group-mean corrected gap-related ERP waveforms (**a**) and the difference of 150 ms, 250 ms and 650 ms gaps and “gap only” events (**b**) measured at the electrode cluster centered on FCz and in the average mastoid signal in the younger and older adult groups. The grey bands indicate the time windows in which the amplitude-related statistical analyses were conducted.

**Figure 2:**
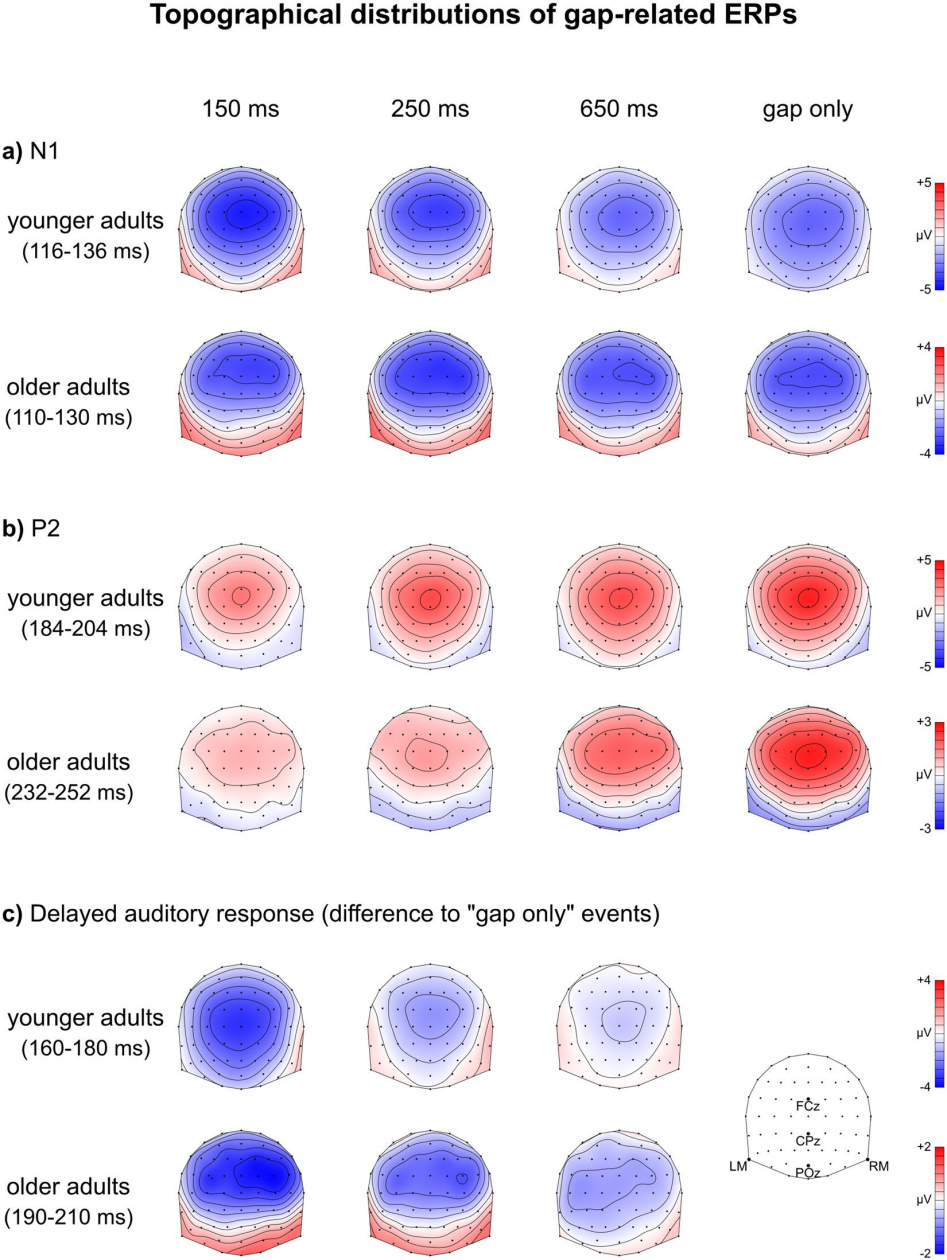
Topographic distributions of the group-mean ERP effects. Topographies of group-mean, corrected gap-related ERPs in the N1 (**a**) and P2 (**b**) time windows and the delayed auditory response overlapping them (**c**). The amplitude scales differ between rows to allow between-group topographical shape comparisons, while allowing the observation of Gap Type amplitude differences for the two groups.

The group-averaged corrected waveforms are presented in Fig. 1a and their topographic distributions are depicted in Fig. 2. All corrected gap-related ERP waveforms showed a negative peak, followed by a positive deflection, which were identified as the frontal aspect of N1 and P2 respectively. For “gap only” events, in the younger adult group, N1 reached its maximum (negative) peak at Cz at 126 ms. P2 peaked at FCz at 194 ms. In older adults, both N1 and P2 peaked at FCz, with 120 and 242 ms latency, respectively. The positive aspect of the N1 peaked at the mastoids slightly earlier than the frontally negative aspect (110 ms in younger and at 106 ms in older adults). The “gap only” N1 amplitudes measured fronto-centrally did not significantly differ between the two groups. The positive aspect of the N1 (i.e. the amplitude measured in the average mastoid signal) was, however, significantly higher (more positive) in the younger adult group (t[43.965] = 3.939, p < 0.001).

**Figure 3:**
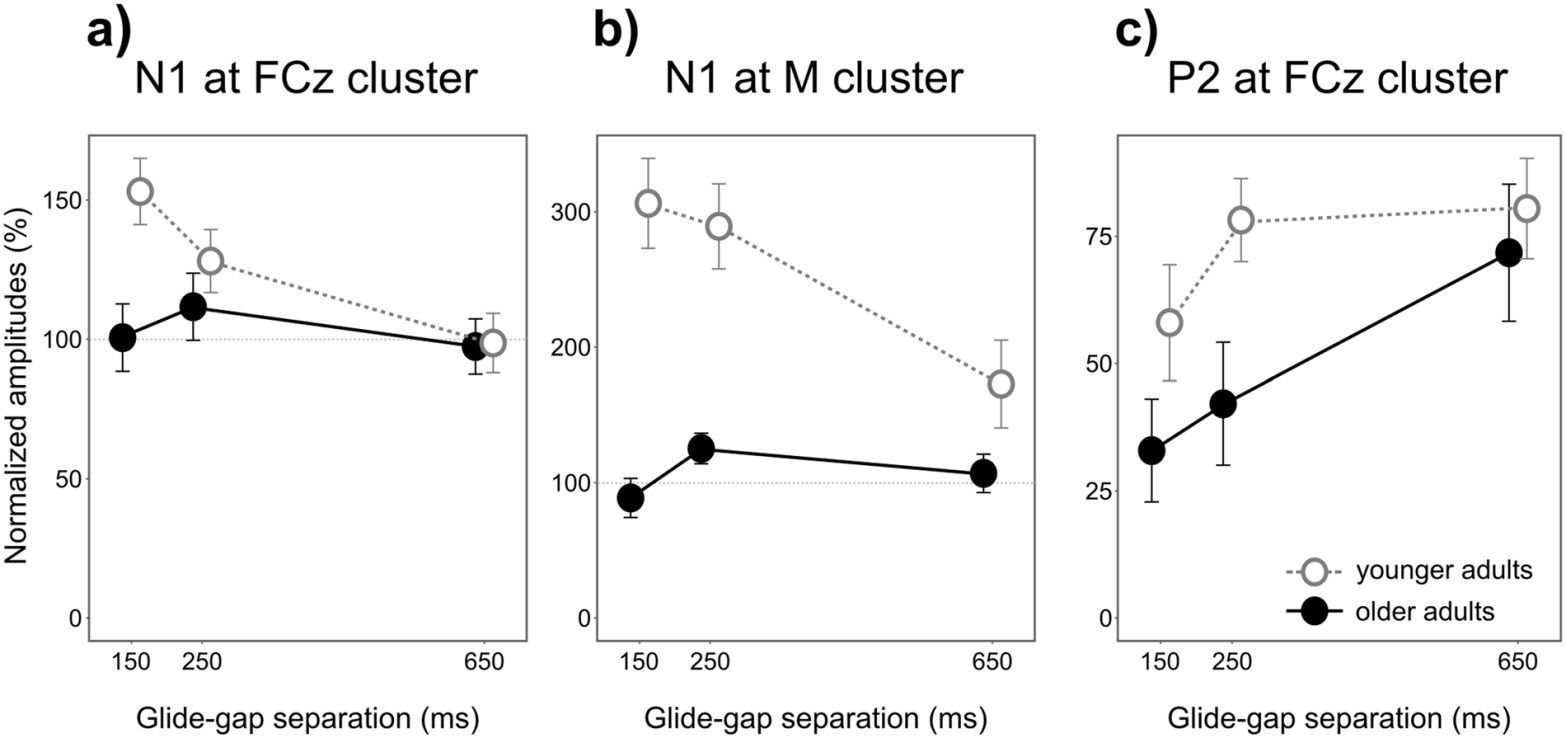
Normalized gap-related ERP amplitudes. Group-mean normalized gap-related ERP amplitudes of measured in the N1 interval at the electrode cluster centered on FCz (**a**) and in the average mastoid signal (**b**), and also in the P2 interval at the electrode cluster centered at FCz (**c**). Whiskers indicate standard errors of means. The basis of the normalization (100% on the vertical axes) refers to the group-mean amplitude in the corresponding corrected “gap only” waveforms.

The one-way Gap Type ANOVAs (150, 250, and 650 ms gap trial) of the fronto-centrally measured N1 amplitudes conducted separately in the two groups showed no significant effect in the older adult group; in younger adults, however a significant Gap Type effect was found: F[3, 66] = 9.531, p < 0.001, η^2^_G_ = 0.159. Follow-up paired Student’s t-tests revealed that the amplitudes were higher (more negative) for the 150 ms than for the 650 ms gaps (t[22]) = 4.349, p < 0.001) or for the “gap only” events (t[22] = 4.762, p < 0.001); amplitudes for the 250 ms gap were also higher (more negative) than for “gap only” events (t[22] = 2.419, p = 0.024). Similarly, the one-way Gap Type ANOVAs of the mastoid signals showed no significant effect in the older adult group, but a significant Gap Type effect was present (F[3, 66] = 17.999, p < 0.001, η^2^_G_ = 0.26) in the younger adult group. The amplitudes for 150, 250, and 650 ms gaps were significantly higher (more positive) than for gap only events (t-scores > 2.559, p-values < 0.019). Significant differences were also present between 150 ms and 650 ms gaps (t[22] = 3.615, p = 0.002), as well as between 250 ms and 650 ms gaps (t[22] = 3.955, p < 0.001). For the normalized N1 amplitudes measured fronto-centrally (Fig. 3a), the Group × Gap Type (150 ms/250 ms/650 ms) ANOVA showed significant Gap Type main effect: F[2, 88] = 5.54, p = 0.005, η^2^_G_ = 0.05; and Group × Gap Type interaction: F[2, 88] = 4.24, p = 0.017, η^2^_G_ = 0.039. Follow-up t-tests between Gap Types showed no significant differences in the older adult group, but in the younger adult group amplitudes were significantly higher for 150 ms than for 650 ms glide-gap separations (t[22] = 4.345, p < 0.001).

The Group × Gap Type ANOVA of the normalized amplitudes for the positive aspect of the N1 (measured in the average mastoid signal) (Fig. 3b) showed a significant Group main effect: F[1, 44] = 29.255, p < 0.001, η^2^_G_ = 0.29; a significant Gap Type main effect (F[2, 88] = 7.6, p < 0.001, η^2^_G_ = 0.06; and a significant Group × Gap Type interaction: F[2, 88] = 8.47, p < 0.001, η^2^_G_ = 0.07). Follow-up t-tests revealed that in younger adults both 150 ms and 250 ms gaps elicited higher amplitudes than 650 ms gaps (t[22] = 3.615, p = 0.002 and t[22] = 3.955, p < 0.001, respectively). In the older adults, however, only one comparison showed a significant difference, and it was in the opposite direction: 150 ms gaps elicited lower amplitudes than 250 ms gaps (t[22] = 2.2, p = 0.04).

For the “gap only” events P2 was significantly larger (more positive) in younger, than in older adults (Welch’s t[43.841] = 2.973, p = 0.005). The one-way Gap Type ANOVA showed significant effects both in the younger (F[3, 66] = 6.553, p < 0.001, η^2^_G_ = 0.098) and in the older adult group (F[3, 66] = 13.672, p < 0.001, η^2^_G_ = 0.174). In the younger adult group, P2 amplitudes to 150 ms gaps were lower than those to 650 ms gaps (t[22] = 2.61, p = 0.016) or “gap only” events (t[22] = 4.472, p < 0.001). Furthermore, 250 ms gaps also resulted in lower amplitudes than “gap only” events (t[22] = 2.373, p = 0.027). In older adults, amplitudes differed between all gap types (t-values > 2.536, p values < 0.017), except for the 150 and 250 ms gaps.

The Group × Gap Type ANOVA of the normalized P2 amplitudes (Fig. 3c) showed a significant Gap Type main effect (F[2, 88] = 8.21, p < 0.001, η^2^_G_ = 0.06) only. Follow-up t-tests showed (with pooled groups) that the modulation of P2 amplitude was significantly larger at 150 ms (t[45] = 4.302, p < 0.001) and 250 ms (t[45] = 2.058, p = 0.045) glide-gap separations in comparison to 650 ms gaps.

In summary, the hypothesis-driven analysis revealed that with shorter glide-gap separation, a stronger N1-enhancement was present (i.e. amplitudes were shifted in the negative direction at fronto-central and in the positive direction at mastoid sites) in the younger, but not in the older adult group. Interestingly, P2 (measured fronto-centrally) was reduced (i.e. amplitudes were shifted in the negative direction) as glide-gap separation decreased in both groups.

### Gap-related ERPs – Exploratory results

The pattern of results presented above opens up the possibility that the observed effects are not due to the modulation of the N1 or P2 components, but rather, they may reflect an overlapping ERP. Indeed, the visual inspection of the waveforms (Fig. 1b) suggests that in the younger adult group the fronto-central N1 enhancement and the P2 reduction are caused by a fronto-centrally negative ERP overlapping both components. Similarly, the visual inspection of the group-average older adult ERP suggests that the P2 reduction, that is, the amplitude shift in the negative direction at fronto-central cites, is paralleled by an amplitude shift in the negative direction at the mastoid sites. To better visualize the glide-gap separation effects, the ERP differences between the ERPs to the 150, 250, and 650 ms gaps and the “gap only” ERP were calculated (Fig. 1b). The waveforms in these differences, referred to as *delayed auditory responses* in the following, were most prominently observable at 150 and 250 ms glide-gap separations, between 90-260 ms in younger adults and between 110-330 ms in the older adults, peaking at 170 ms at Cz in the younger, and at 200 ms at FC4 in the older adult group at 150 ms glide-gap separation.

To confirm the visual impression that the amplitude of the delayed auditory response was modulated by glide-gap separation, the average amplitudes in a 20 ms time-window centered at the local minima of the “150 ms gap” – minus – “gap only” difference at the FCz cluster were measured separately for both groups. Similarly, because the polarity of the negativity seemed to be inverted at the mastoids, amplitudes were measured in a 20 ms time-window around the positive peak of “150 ms gap” – minus – “gap only” events waveform, separately for the two age groups. Similarly to the hypothesis-driven analysis, the “250 ms gap” – minus “gap only” and 650 ms gaps – minus – “gap only” amplitudes were normalized by the “150 ms gap” – minus – “gap only” amplitudes, then submitted to Group × Gap Type (250 ms/650 ms) ANOVAs.

At the FCz cluster, the Group × Gap Type (250 ms/650 ms) ANOVA for the normalized amplitudes showed only a significant Gap Type main effect: F[1, 44] = 6.11, p = 0.017, η^2^_G_ = 0.04. The same type of analysis of the mastoid signals showed a significant Gap Type main effect only: F[1, 44] = 1828, p < 0.001, η^2^_G_ = 0.17. In both cases the amplitudes were higher (more negative for the FCz, cluster, and more positive at the mastoids) for the 250 than for the 650 ms gaps.

To confirm the visual impression that there was a latency difference between groups, the latencies of the “150 ms gap” – minus – “gap only” waveforms, as measured with a fractional area technique in combination with a jackknife procedure^57^ were submitted to a Welch t-test. This procedure was also used to compare the latencies of the delayed auditory responses at FCz and its positive aspect at the mastoids as well. When defining the latencies at the averaged mastoids, in case of three participants the boundary defined two areas in the younger adults group. In their case, the larger area was selected, which corresponded to the earlier one. Note that in the following the jackknife-adjusted F-, t- and p-values are reported. The degrees of freedom remained unadjusted.

In the “150 ms gap” – minus – “gap only” ERP difference, a significant group-difference was present both at the FCz-cluster (t[42.405] = 6.823, p < 0.001), and at mastoids (t[24.947] = 4.251, p < 0.001). In both cases, the waveform peaked earlier in the younger adult group. The Group (younger adult/older adult) × Site (FCz cluster/M cluster) ANOVA revealed significant Group (F[1, 44] = 44.909, p < 0.001, η^2^_G_ = 0.996) and Site (F[1, 44] = 18.071, p < 0.001, η^2^_G_ = 0.988) main effects. The Group × Site interaction was not significant.

### Glide-related ERPs

**Figure 4:**
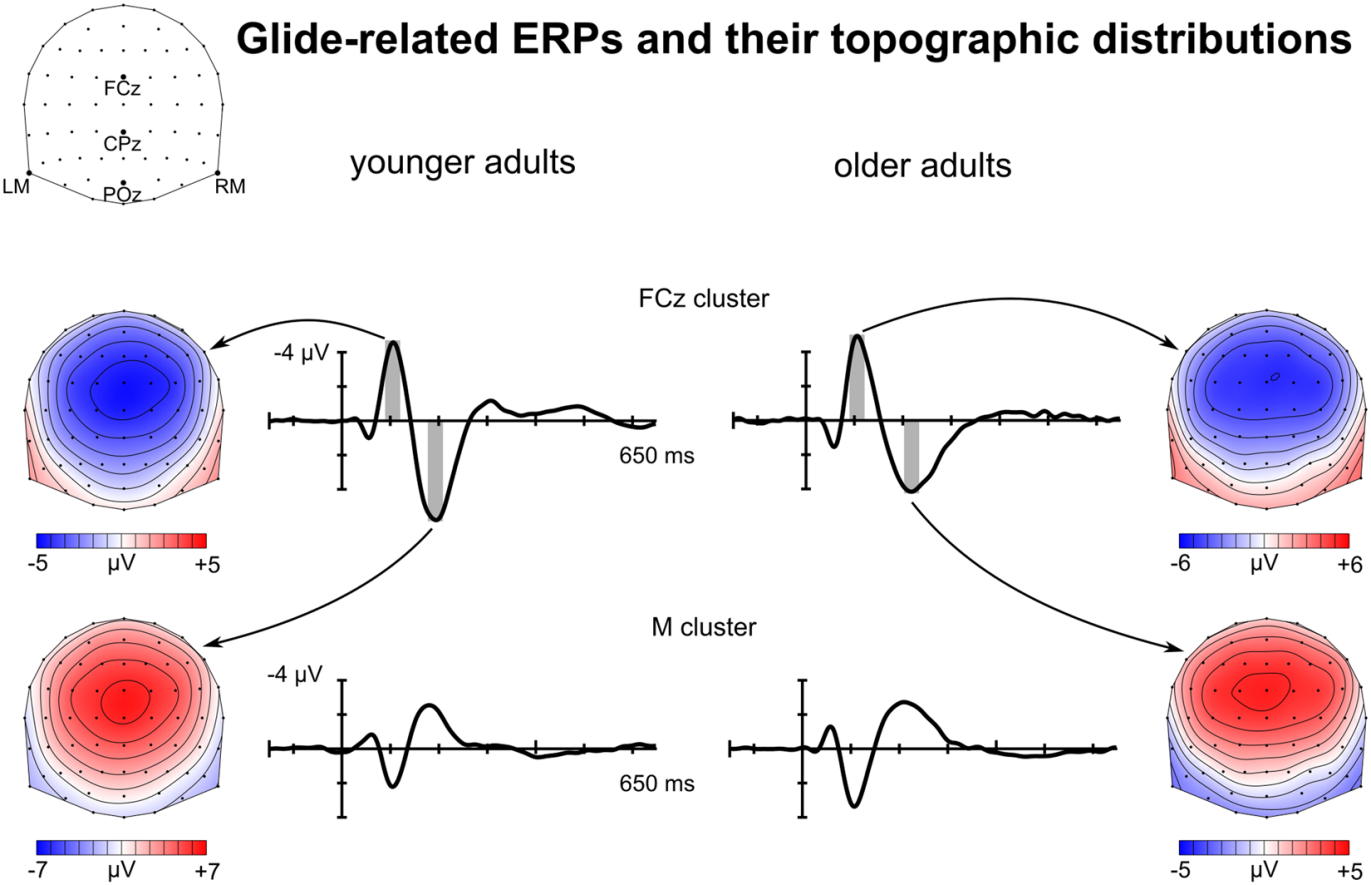
Group-mean glide-related ERPs and the corresponding topographies in the N1 and P2 time ranges in the younger and older adult groups. The grey bands indicate the time windows where the amplitude measurements were performed and for which the topographic distributions are presented. The amplitude scales differ in order to support the comparison of distribution shape information between groups.

Glides elicited a clear N1 and P2 in both groups (Fig. 4). In the younger adult group, N1 peaked at 106 ms and P2 peaked 194 ms, both at FCz, in the group-average waveform. In the older adult group, N1 and P2 reached their maximum peaks at FCz as well; at 106 ms and at 218 ms, respectively. Glide-related amplitudes for N1 and P2 were also compared between the two groups by Welch t-tests at the FCz cluster. There was no significant difference in N1 amplitudes, but P2 was elicited with significantly lower amplitudes in the older than in the younger adult group (t[38.428] = 2.887, p = 0.006).

## Discussion

The goal of the present study was to compare the duration of a distracted state induced by randomly occurring rare, background auditory events between younger and older adults. While participants watched a silent movie with subtitles, continuous tones containing rare glides (distracters) and frequent gaps (probes) were presented. Based on previous studies, we hypothesized that gap-related N1 and P2 ERPs would be enhanced when gaps closely followed glides, reflecting an increased readiness of the auditory system to process incoming stimuli. Based on the hypothesis that older adults showed a higher distractibility, and decreased ability to inhibit the processing of task-irrelevant pieces of information, we hypothesized that such ERP enhancements would be present for longer glide-gap separations in older than in younger adults.

Gaps elicited N1 and P2 with higher amplitudes in the younger than in the older adult group (fitting previous results^55, 56, 58^. Although the results showing an ERP enhancement with decreasing glide-gap separations in the N1 time range in the younger adult group are on a par with previous studies^28, 39–44^, the attenuated amplitudes in the P2 interval, and the comparison with the older adult group suggests a more economical explanation. Instead of a modulation of the N1 and P2 peaks, the hypothesis-driven analyses of the ERP amplitudes in the N1 and P2 time-intervals revealed a pattern which could be best described as an overlap by a fronto-centrally negative waveform with a polarity reversal at the mastoids. This – presumably – auditory response was delayed in comparison to the N1, which resulted in a virtual enhancement of the N1 and attenuation of the P2 in the younger adult group. Because the overlapping waveform was even more delayed in the older adult group, its presence was manifested only in a marked (but virtual) P2 attenuation. The delayed auditory response showed decreasing amplitudes with increasing glide-gap separations.

This result contradicts our hypothesis that rare glides temporarily raised the responsiveness of the auditory system to process closely following events, and thus invalidates the premise of our initial hypothesis regarding an age-related increase in the persistence of such ERP enhancements. In addition, neither the distraction-based hypothesis (that a brief, involuntary allocation of attention to the tone lead to enhanced processing), nor the latent inhibition hypothesis (suggesting that a basic excitatory effect preceding the onset of inhibition enhances N1-elcitation) can explain the emergence of an additional gap-related ERP.

The idea that the higher N1 (and lower P2) amplitude for a tone closely preceded by another tone was not a genuine N1- or P2-modulation, but the result of an overlapping negative ERP component has been suggested by Wang and colleagues^44^. They speculated that the overlapping ERP may have been an MMN, which was elicited because of the relatively short (i.e 100, 200 ms) separations from the preceding tone in comparison to the typical inter-stimulus intervals (sampled from an equiprobable 100-1000 ms distribution, with a mean of 500 ms) used in their study (see e.g. Sable *et al*.^59^). The idea that MMN may contribute to the N1 enhancement was also hinted at by Todd and colleagues^42^ who found that the N1 facilitation to very short (50 ms) inter-tone intervals and the amplitude of the MMN to rare duration deviants (100 ms tones presented among 50 ms tones) were strongly correlated in healthy adults. The present results showing that the delayed auditory response had a polarity inversion at the mastoids also fit the MMN explanation, because – being of supratemporal origin – MMN also often shows a similar inversion^60–62^.

A further possibility is that the closely following gap is processed *together* with the preceding glide by the auditory system. Indeed, numerous studies suggested that processes underlying MMN generation integrate information over longer intervals (150–300 ms, see e.g. Tervaniemi *et al*.^63^, Yabe *et al*.^64^, Grimm *et al*.^65^). The temporal window of integration seems to be of similar duration between younger and older adults^66^. Although the glide probably elicited an enhanced N1 and possibly an MMN^27^ in itself, the closely following gap may be integrated with the glide, and treated as a single unit of stimulation. Because the glide-and-gap event-combination was as rare as the glide-only event in the present paradigm, an MMN time-locked to the gap may additionally be elicited.

Studies investigating age-related MMN latency differences in conditions of inattention for tones deviating in pitch, duration, or novelty from the frequently presented tones found either no differences^52, 67, 68^ or age-related delays^69, 70^ (a tendency: Gaeta *et al*.^71^). MMNs elicited by unattended, rare tones with gaps showed age-related delays^55, 72^. The fact that the delayed auditory response was elicited later in older than in younger adults is therefore also compatible with the notion that the ERP is a gap-related MMN. Although the gap-duration was longer than that used in the studies by Alain *et al*.^55^ and Bertoli *et al*.^72^, and it allowed close-to-perfect gap-detection rates in an active version of the administered paradigm for both younger and older adults^58^, the age-related latency-difference may still be the consequence of an age-related deterioration of fine temporal resolution^73^.

In summary, the present results confirmed previous ERP findings showing enhanced ERP responses to auditory events shortly following another auditory event. In contrast to previous studies, by comparing younger and older adults, the present study provided evidence that the enhanced auditory ERP response was not due to the enhancement of the N1, but to an overlapping ERP originating from the auditory cortex, presumably an MMN. The delayed elicitation of this waveform suggests that central auditory processes related to the detection of gaps in continuous tones are slowed in older adults.

## Methods

### Participants

50 healthy adult women participated in the study, 25 persons in the younger and 25 in the older adult group. Younger adults were recruited by a student part-time job agency; older adults were recruited from the department’s participant database. All of them were compensated by modest amounts of money. Due to excessive amounts of movement artifacts, only the data from 46 participants was used for further analyses. The final sample consisted of 23 younger (3 left-handed) and 23 older (all right handed) adults. The average age was 22.13 years (SD = 2.01; from 18 to 26 years) in the younger and 68 years (SD = 3.71; from 62 to 76 years) in the older adult group. Participants gave written informed consent. The experiment was conducted in accordance with the Declaration of Helsinki and the protocol was approved by the United Ethical Review Committee for Research in Psychology (Hungary).

All participants reported correct or corrected-to-normal vision and normal hearing. Older adults had higher hearing thresholds than younger adults in the 250–2000 Hz frequency range (as assessed by an SA-6 audiometer, MEDIROLL, Debrecen, Hungary, see Table 1). The threshold difference between the two ears was not higher than 25 dB at any of the frequencies. In order to compensate for potential hearing differences between participants, the intensity of the sounds presented in the experiment was individually adjusted to 50 dB above the 75% hearing threshold measured by the Single Interval Adjustment Matrix procedure^74^; see also Shepherd *et al*.^75^.

**Table 1:** Group mean hearing thresholds (dB SPL) and the corresponding standard deviations in the two groups in the 250–2000 Hz range.

Group	250 Hz	500 Hz	1000 Hz	2000 Hz
Younger	13.37 (±4.6)	10.11 (±6.45)	3.04 (±5.22)	4.45 (±6.69)
Older	19.89 (±7.99)	17.93 (±7.57)	12.5 (±8.34)	20.97 (±12.96)
	t = 4.80, p < 0.001	t = 5.34, p < 0.001	t = 6.51, p < 0.001	t = 7.78, p < 0.001

The Hungarian version of Wechsler Intelligence Scale (WAIS-IV^76^) was administered in a separate session to exclude dementia-related differences between the two age-groups. The mean IQ scores were 119.78 (SD = 18.07; from 85 to 156) in the older, and 106.13 (SD = 18.05; from 81 to 150) in the younger adult group, showing a significant difference (t[44] = 2.564, p = 0.014). Moreover, while the IQ scores of younger adults was average (t[22] = 1.629, p = 0.118), older adults were characterized with significantly higher IQ than the population average (t[22] = 5.252, p < 0.001).

### Stimuli and procedure

**Figure 5:**
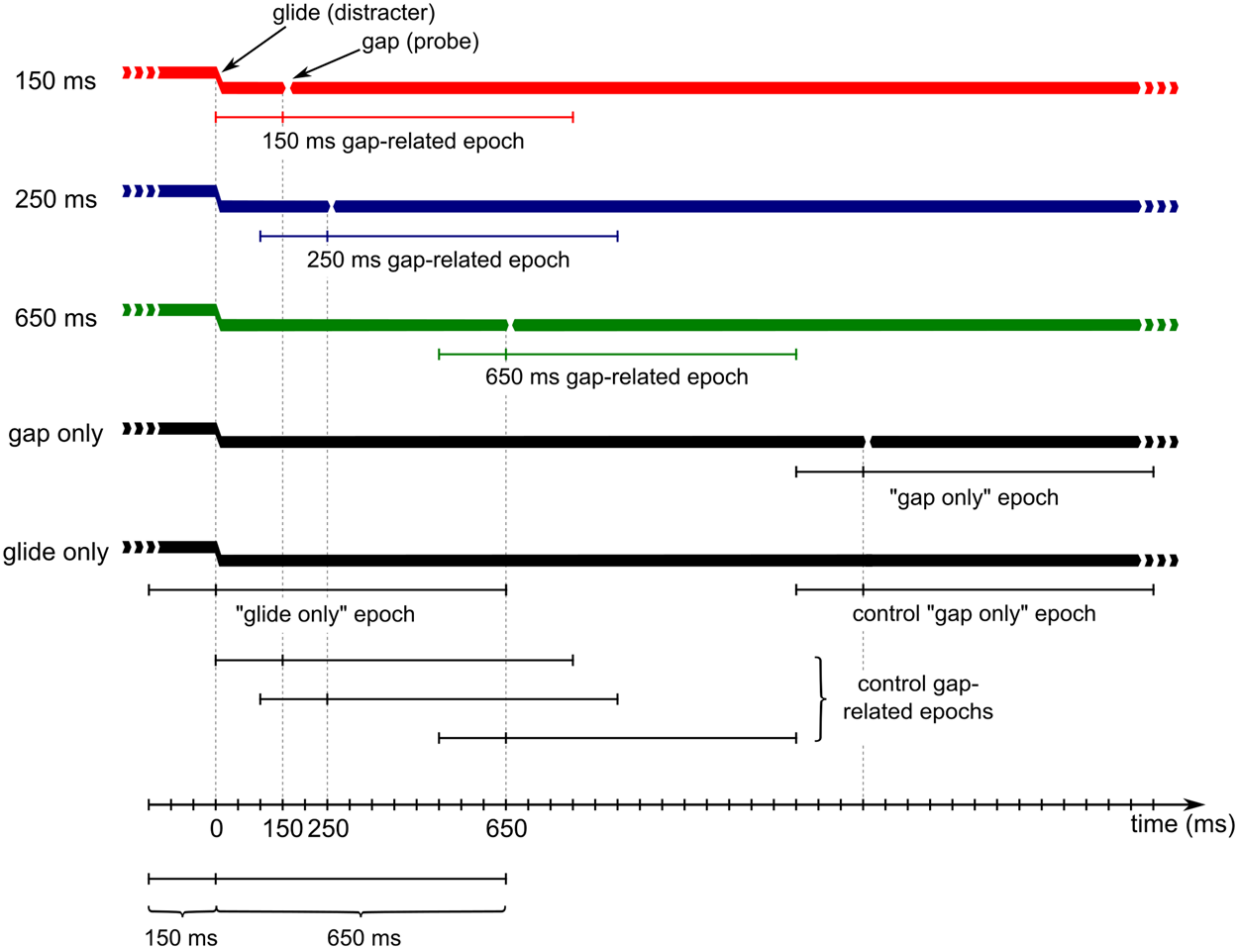
The schematic design of the study. Thick lines represent continuous tones, with vertical displacements indicating glides. Glide- and gap-related (150 ms, 250 ms, 650 ms and “gap only”) epochs and the corresponding control epochs are indicated below the tones by intervals markings.

During the experiment, participants were sitting in an armchair in an electrically shielded and acoustically isolated room, and watched a self-selected movie with subtitles (but without sound) while continuous, 331 s long tones were presented through Sennheiser (HD-600, Sennheiser, Wedemark, Germany) headphones. Participants were instructed to watch the movie and ignore auditory stimuli. The tones were generated using Csound (version 5.17.11, www.csounds.com), with a sampling rate of 44.1 kHz and consisted of three harmonics: the fundamental, second and third harmonics (the first one was missing). Each harmonic was presented with the same amplitude. The tone was alternating between two pitches (characterized by 220 Hz and 277 Hz base frequencies) by quick, 10 ms long glides (*glissandos*). Such glides could occur at fixed timepoints separated by 1300 ms. Glides could occur at these timepoints with a 1/7 probability, with the constraint that consecutive glides had to be separated by at least 3900 ms. Thus, on average, 36 glides occurred in each continuous tone (i.e. in each recording block). The tones also contained short gaps with a 10 ms silent period and 10-10 ms linear fall and rise times. Such gaps were randomly inserted with a probability of 50% after timepoints at which glides could occur. When a gap was inserted, it followed the potential glide timepoint by 150, 250 or 650 ms (with equal probability). In the following, we refer to gaps following an actual glide in 150, 250, or 650 ms as “150 ms gap”, “250 ms gap”, and “650 ms gap” events (the design is shown in Fig. 5). Gaps not following a glide within 650 ms (i.e. gaps for which no glide occurred at the preceding timepoint) are referred to as “gap only” events. Glides which were not followed by any gaps in 650 ms, referred as “glide only” events. 14 tones (i.e. blocks) were presented during the experiment, which were separated by short breaks as needed. After the 7^th^ block, a longer break could be taken depending on the participants’ preference.

Before the experiment, 2–3 minutes long EEG-recording was also taken to capture eye-movement-related EEG-activity (with instructions as described by Schlögl *et al*.^77^).

### EEG recording

The continuous EEG was recorded at a sampling rate of 500 Hz using a Neuroscan Synamp 2 (Compumedics Inc., Victoria, Australia) amplifier from 61 Ag/AgCl electrodes were mounted on an EasyCap (EASYCAP GmbH, Herrsching, Germany) arranged by the 10% system^78^. Two additional electrodes were placed at mastoids. The reference and the ground electrodes were placed at the tip of the nose and to the forehead, respectively. Horizontal electro-oculogram was measured by electrodes attached near the outer canthi of the eyes while the vertical electro-oculogram was calculated offline as the difference of Fp1 electrode and an electrode placed under the left eye.

Continuous EEG data was filtered offline using first a 1 Hz highpass filter (Kaiser-windowed sinc finite impulse response filter, beta of 4.53, 2929 coefficients; 0.5 Hz transition bandwith, stopband attenuation at least 50 dB). After that, an eye movement correction procedure was applied as described by Schlögl and colleagues^77^. Finally, the corrected EEG data was filtered again, using a 30 Hz lowpass filter (Kaiser-windowed sinc finite impulse response filter, beta of 4.53, 2929 coefficients; 0.5 Hz transition bandwidth, stopband attenuation at least 50 dB).

The EEG was segmented into 800 ms long epochs corresponding to the 150 ms gap, 250 ms gap, 650 ms gap, and “gap only” events, including a 150 ms long pre- and a 650 ms post-stimulus interval. To eliminate non-gap-related ERP-contributions, further EEG segments were extracted in which gaps could potentially occur (i. e. 150 ms, 250 ms and 650 ms after the onset of potential glide timepoints and after the onset of glides which were not followed by any gaps in 650 ms). These segments are referred to control epochs in the following. After discarding epochs with a signal range exceeding 100 µV on any channel, the control ERPs were subtracted from the corresponding ERPs of the 150 ms, 250 ms and 650 ms gaps and “gap only” events (see Fig. 5). The results of these subtractions are referred to as *corrected* waveforms. Glide-related ERPs were also investigated for “glide only” events. The datasets generated during and/or analysed during the current study are available from the corresponding author on reasonable request.

### Statistical analysis

The analysis of gap-related ERPs consisted of a hypothesis-driven, and an explorative part. In the hypothesis-driven part, gap-related N1 waveforms were identified for 150 ms, 250 ms, 650 ms gap, and “gap only” events in the group-averaged corrected waveforms, separately in the two groups. Individual ERP amplitudes were calculated in 20 ms long windows centered at the N1 peak latency measured in the group-averaged corrected waveforms for “gap only” events. In order to improve signal-to-noise ratio, statistical analyses were conducted at a fronto-central cluster of electrodes including FCz, Cz, Fz, FC1 and FC2 (referred to as FCz-cluster in the following). The mastoid signals were also averaged, this average signal is labeled M. To assess whether gap-related N1 amplitudes *per se* differed between groups, gap only N1 amplitudes measured in the two groups were compared by Welch’s t-tests. Then N1 amplitudes elicited in the two groups by different Gap Types were submitted separately to repeated measures ANOVAs. To compare the *modulation* of the N1 amplitude by glide-gap separation between groups, the 150 ms, 250 ms and 650 ms gap amplitudes were normalized by the “gap only” N1 amplitudes separately in the two age groups. These normalized amplitudes were submitted to a Group (older/younger) × Gap Type (150 ms/250 ms/650 ms) mixed ANOVA. These analyses were also performed for the positive aspect of the N1 measured in the averaged mastoid signal, as well as for the P2 amplitudes measured at the FCz-cluster.

The visual inspection of the ERP waveforms suggested that the amplitude differences found in the hypothesis-driven part of the analysis were not caused by pure modulations of the N1 or P2 waveforms, but by the emergence of a fronto-centrally negative deflection overlapping both of these waveforms. Similarly to N1, its amplitude also seemed to be modulated by the glide-gap separation and its polarity was inverted at the mastoids. Because of these attributes, in the following the deflection is referred to as *delayed auditory response*. Therefore, in the *explorative* part of the analyses, three difference waveforms were calculated by subtracting the ERP to the corrected gap only events from the ERP to the 150, 250 and 650 ms gap events to characterize this deflection. Since the 150 ms – minus – “gap only” difference waveform showed the highest (negative) amplitude, we normalized the amplitudes of the 250 ms – minus – “gap only” and 650 ms – minus – “gap only” waveforms separately in each group in a 20 ms window centered at this peak. The mean amplitudes were calculated at FCz cluster, and were submitted to a Group (older/younger) × Gap Type (250 ms/650 ms) ANOVA.

From the visual inspection of the group-average difference waveforms, it was also apparent that the delayed auditory response emerged later in the older than in the younger adult group. To verify this post-hoc observation, the latencies for the 150 ms gap – minus – “gap only” waveforms in the two groups were compared by Welch’s t-tests following the jackknife procedure combined with a fractional area technique based on Kiesel *et al*.’s^57^ description. The latencies were determined separately for the two groups, using a boundary of −0.5 µV at the FCz-cluster. Since it seemed to be inverted at the mastoid sites, we also measured its positive aspect in the average mastoid signal with a 0.2 µV boundary. Latencies were defined as the halving points of the area between 50 and 300 ms in the younger adults and between 50 and 400 ms in the older adults. To further compare the temporal and topographical characteristics of this effect, a Group (younger adults/older adults) × Site (FCz cluster/M cluster) ANOVA was applied.

Finally, N1 and P2 amplitudes elicited by “glide only” events were also compared between the older and the younger adult group. The individual ERPs were averaged in a 20 ms time window centered at the group-mean negative peak, separately for the two age groups. The N1 and P2 amplitudes measured at the FCz-cluster were analyzed in Welch’s t-tests. All statistical tests were calculated in R (version 3.1.0, R Core Team, 2014). Generalized eta squared effect sizes^79, 80^ are also reported.
